# The association between unexpected weight loss and cancer diagnosis in primary care: a matched cohort analysis of 65,000 presentations

**DOI:** 10.1038/s41416-020-0829-3

**Published:** 2020-04-15

**Authors:** Brian D. Nicholson, Willie Hamilton, Constantinos Koshiaris, Jason L. Oke, F. D. Richard Hobbs, Paul Aveyard

**Affiliations:** 1 Nuffield Department of Primary Care Health Sciences, University of Oxford, Oxford, OX2 6GG UK; 2 Medical School, University of Exeter, Exeter, UK

**Keywords:** Signs and symptoms, Diagnosis, Health policy

## Abstract

**Background:**

We aimed to understand the time period of cancer diagnosis and the cancer types detected in primary care patients with unexpected weight loss (UWL) to inform cancer guidelines.

**Methods:**

This retrospective matched cohort study used cancer registry linked electronic health records from the UK’s Clinical Practice Research Datalink from between 2000 and 2014. Univariable and multivariable time-to-event analyses examined the association between UWL, and all cancers combined, cancer site and stage.

**Results:**

In all, 63,973 patients had UWL recorded, of whom 1375 (2.2%) were diagnosed with cancer within 2 years (days-to-diagnosis: mean 181; median 80). Men with UWL (HR 3.28 (2.88–3.73)) and women (1.87 (1.68–2.08)) were more likely than comparators to be diagnosed with cancer within 3 months. The association was greatest in men aged ≥50 years and women ≥70 years. The commonest cancers were pancreas, cancer of unknown primary, gastro-oesophageal, lymphoma, hepatobiliary, lung, bowel and renal-tract. The majority were late-stage, but there was some evidence of association with stage II and stage III cancers. In the 3–24 months after presenting with UWL, cancer diagnosis was less likely than in comparators.

**Conclusion:**

UWL recorded in primary care is associated with a broad range of cancer sites of early and late-stage.

## Introduction

Many developed countries have national strategies to improve survival from cancer. A key component is diagnosing cancer earlier.^1^ National guidelines encourage primary care clinicians/general practitioners (GPs) to act on ‘alarm’ symptoms, such as haemoptysis or rectal bleeding, and to refer patients with these symptoms rapidly for specialist investigation.^2,3^ However, half of patients with cancer present with non-specific symptoms, such as unexpected weight loss, that do not point to a specific cancer site.^4,5^ These patients have longer times to diagnosis, are less likely to be referred urgently, and more likely to be diagnosed as an emergency or at an advanced stage.^6–10^ Given that investigations for possible cancer in these patients need to be wider-ranging than for localising symptoms,^11,12^ perhaps with bespoke clinical services,^13,14^ we need to understand which cancers are diagnosed in patients presenting to primary care with unexpected weight loss.

A systematic review from 2018 found 25 primary care studies reporting a positive association between presenting to primary care with unexpected weight loss and a subsequent diagnosis of 10 different cancers.^15^ The sensitivity of unexpected weight loss for cancer ranged from 2% to 47%, the specificity from 92% to 99%. None of the studies reported on cancer stage; knowing this is crucial when considering how to improve outcomes. The review may have incorrectly estimated the association between unexpected weight loss and cancer as for some cancer sites no evidence was available. Moreover, some study designs in the review were affected by bias. For example, sensitivity was higher in studies at risk of recall bias and positive predictive values were higher in case–control studies compared with cohort studies on the same tumour site. Furthermore, most studies examined the association between unexpected weight loss and a diagnosis of cancer within the next 2 years, but it is plausible that the association between unexpected weight loss and cancer diagnosis changes over time.^16^

Therefore, since the current evidence on this topic is limited and conflicting, we aimed to examine the association between unexpected weight loss and a diagnosis of cancer, and whether any association varies over time, for all cancers combined and separately by cancer site and cancer stage.

## Methods

### Study design and population

**Fig. 1:**
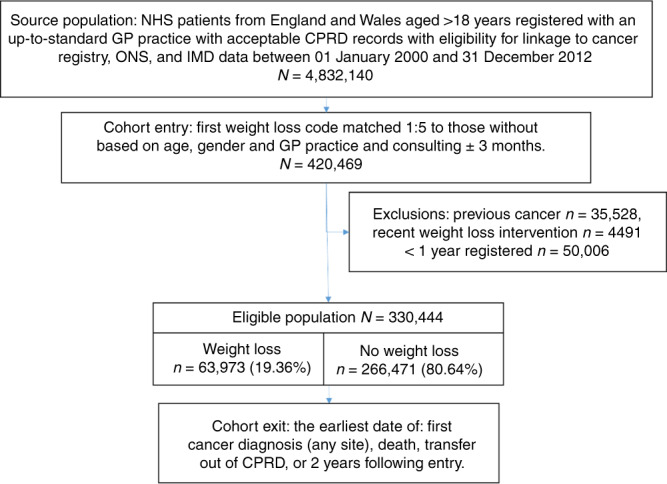
Study population flow diagram. This flow diagram details the source population, cohort entry criteria, exclusion criteria, the frequency of the eligible population with and without unexpected weight loss, and the study exit criteria.

This was a retrospective matched cohort study using electronic health records (EHR) from the Clinical Practice Research Datalink (CPRD): a representative anonymised primary care records database covering 6.9% of the UK population.^17^ After acceptance by CPRD’s Independent Scientific Advisory Committee (16_164A2A) a protocol was published,^18^ to which we added sensitivity analyses. Cohort entry, exclusions and exit are summarised in Fig. 1.

#### Patients with and without unexpected weight loss

We identified which unexpected weight loss codes that were truly associated with objectively measured weight loss in a previous internal validation study and used these to define unexpected weight loss in this study^19^ (Supplementary information 1). Five patients of the same year of birth and sex who had consulted in the same practice within 3 months of the patient with unexpected weight loss were matched as a comparator to each patient with coded unexpected weight loss. We then excluded cases and comparators registered within the previous year, prescribed a weight loss medication or who had bariatric surgery in the previous 6 months, plus patients with a previous diagnosis of cancer. If a patient with unexpected weight loss was excluded, their matched comparators were excluded. If a comparator patient without unexpected weight loss was excluded, we excluded only that one comparator.

#### Identifying patients with subsequent diagnosis of cancer

The records were examined for a diagnosis of cancer for 2 years after the index date. Cancer diagnoses were identified using codes in CPRD and codes for all ICD-O categories in the linked cancer registry data. We grouped cancers together typically investigated together. For example, renal, ureteric and bladder cancer as renal tract, and liver, gallbladder and biliary tree as hepatobiliary (HPB).^18^ The first cancer code following the index date was used to define cancer type. We classified cancer of unknown primary (CUP) if patients’ records indicated secondary cancer but no primary. Cancer stage was analysed by individual stage, and then grouped as early-stage (I and II) and late-stage (III and IV).

#### Covariates

Potential confounders were taken from the dataset before the index date as defined in Table 1. Missing data was categorised using a missing indicator variable as shown in Table 1. We also adjusted for comorbidity that could cause weight loss as defined in our previous study of weight recording in primary care.^20^ All code lists are available from the corresponding author.

**Table 1:** Baseline demographics of the study population by unexpected weight loss (UWL) status.

		UWL	No UWL	Chi^2^
	*N*	*n* (%)	*n* (%)	*p*-value
Total	330444	63973 (19.36)	266471 (80.64)	n/a
Gender				
Male	140002	26758 (41.83)	113244 (42.50)	<0.001
Female	190442	37215 (58.17)	153227 (57.50)	<0.001
Mean age (years)	330444	58.66 (SD 20.98)	59.01 (SD 20.56)	n/a
Median age (years)	330444	61 (IQR 42-77)	61 (IQR 43-77)	n/a
Age-group (years)				
18–39	69837	14290 (22.34)	55547 (20.85)	<0.001
40–49	42159	8016 (12.53)	34143 (12.81)	0.155
50–59	45229	8511 (13.30)	36718 (13.78)	0.018
60–69	48292	9017 (14.10)	39275 (14.73)	0.003
70–79	61125	11565 (18.08)	49560 (18.60)	0.023
80+	63828	12574 (19.66)	51254 (19.23)	0.067
Smoking				
Current	33440	9629 (15.05)	23811 (8.94)	<0.001
Ex-Smoker	38153	7164 (11.10)	30989 (11.63)	<0.001
Non-Smoker	84913	14457 (22.60)	70456 (26.44)	0.410
Missing	173938	32723 (51.15)	141215 (52.99)	<0.001
Alcohol				
Current	99340	18435 (28.82)	80905 (30.36)	<0.001
Non-Drinker	35950	8095 (12.65)	27855 (10.45)	<0.001
Past-Drinker	4015	1087 (1.70)	2928 (1.10)	<0.001
Missing	191139	36356 (56.83)	154783 (58.09)	<0.001
Family history cancer				
Yes	19491	4119 (6.44)	15372 (5.77)	<0.001
No	310953	59854 (93.56)	251099 (94.23)	<0.001
IMD quintile				
I	72623	12552 (19.59)	60071 (22.56)	<0.001
II	72525	13212 (20.67)	59313 (22.28)	<0.001
III	70486	13507 (21.13)	56979 (21.40)	0.135
IV	59757	12156 (19.18)	47601 (17.88)	<0.001
V	54694	12417 (19.43)	42277 (15.88)	<0.001
Comorbidity				
0	82373	8870 (13.87)	73503 (27.58)	<0.001
1	74057	12765 (19.95)	61292 (23.00)	<0.001
2	59361	12641 (19.76)	46720 (17.53)	<0.001
3	43747	10378 (16.22)	33369 (12.52)	<0.001
4	29756	7638 (11.94)	22118 (8.30)	<0.001
5+	44153	11684 (18.26)	29469 (11.06)	<0.001
BMI group				
Underweight	11294	6691 (10.88)	4603 (1.73)	<0.001
Normal	122298	33846 (52.91)	88452 (33.19)	<0.001
Overweight	95490	10790 (16.87)	84700 (31.79)	<0.001
Obese	56968	5141 (8.04)	51827 (19.45)	<0.001
Missing	44124	7235 (11.31)	36889 (13.84)	<0.001

*SD* standard deviation, *IQR* inter-quartile range.

### Statistical analysis

#### Time to event analyses

The number of days between the index date and the cancer diagnosis is defined as the diagnostic interval (DI).^21^ It was summarised using the median (inter-quartile range (IQR)) and mean (standard deviation (SD)) for cases and comparators, and differences between medians assessed using the k-sample test. We identified whether unexpected weight loss was associated with being diagnosed with cancer for all cancers combined and separately by cancer type, cancer stage, by gender and by age-group. To do so, we plotted and compared smoothed hazard and cumulative hazard rates, over time from the index consultation, assessing significance using the Wilcoxon rank test. Multivariable Cox models derived hazard ratios (HRs) for cancer for each 6-month period of follow-up, adjusted for all covariates as detailed in Table 1. We chose to do this a priori to explore the time varying nature of the predictive value for cancer diagnosis. We assessed whether these models met the proportional hazards assumption using the Schoenfeld residuals test (SRT).^22^ The SRT failed for cancer overall in both genders and lung cancer in men in the 6-month models (Table 2). After refitting Cox models with an initial 3-month interval and stratifying the analysis by comorbidity, all cancers passed the SRT. Adjusted HRs for 0–3 m and 0–6 m intervals were plotted in order of magnitude, and by gender.

**Table 2:** Proportion of cancers diagnosed by cancer site and cancer stage and hazard ratios (HRs) for a diagnosis of cancer in patients presenting to primary care with unexpected weight loss (UWL).

	Men				Women			
	0–3 months of follow-up		0–6 months of follow-up		0–3 months of follow-up		0–6 months of follow-up	
Cancer site	*n* (%)	UWL HR (95% CI)	*n* (%)	UWL HR (95% CI)	*n* (%)	UWL HR (95% CI)	*n* (%)	UWL HR (95% CI)
All cancers	893 (100)	3.28 (2.88–3.73)^a^	1565 (100)	1.87 (1.68–2.08)^a,b^	787 (100)	1.85 (1.6–2.14)^a^	1454 (100)	1.12 (0.99–1.26)^b^
Bone connective and soft tissue	20 (2.2)	4.58 (1.87–11.18)^a^	43 (2.7)	1.97 (1.01–3.87)^c^	16 (2.0)	1.96 (0.66–5.82)^c^	42 (2.9)	0.92 (0.37–2.28)
Bowel	123 (13.8)	2.56 (1.78–3.67)^a^	202 (12.9)	1.49 (1.09–2.03)^a^	119 (15.1)	1.55 (1.07–2.25)	208 (14.3)	1.10 (0.81–1.50)
Breast					144 (18.3)	0.13 (0.05-0.31)^a^	318 (21.9)	0.07 (0.03–0.16)^a^
Cancer of unknown primary	46 (5.2)	8.20 (4.41–15.26)^a^	77 (4.9)	4.29 (2.69–6.83)^a^	37 (4.7)	4.22 (2.24–7.96)^a^	64 (4.4)	2.10 (1.26–3.50)^a^
Central nervous system	9 (1.0)	0.32 (0.04–2.53)	18 (1.2)	0.23 (0.03–1.71)	21 (2.7)	1.34 (0.48–3.75)	37 (2.5)	1.27 (0.59–2.76)
Gastro-oesophageal	93 (10.4)	7.10 (4.66–10.81)^a^	123 (7.9)	4.26 (2.97–6.1)^a^	48 (6.1)	5.45 (3.09–9.63)^a^	81 (5.6)	2.66 (1.72–4.13)^a^
Head and Neck	15 (1.7)	0.58 (0.17–2.02)	37 (2.4)	0.51 (0.21–1.23)	12 (1.5)	0.29 (0.04–2.28)	21 (1.4)	0.35 (0.08–1.53)
Hepatobiliary	28 (3.1)	5.26 (2.63–10.52)^a^	48 (3.1)	3.85 (2.19-6.76)^a^	22 (2.8)	5.36 (2.4–11.95)^a^	30 (2.1)	4.27 (2.13–8.55)^a^
Leukaemia	21 (2.4)	1.25 (0.49–3.22)	36 (2.3)	1.03 (0.48–2.19)	13 (1.7)	0.20 (0.03–1.48)	35 (2.4)	0.29 (0.09–0.96)^c^
Lung	185 (20.7)	5.93 (4.43–7.94)^a^	306 (19.6)	2.74 (2.18–3.43)^a,b^	107 (13.6)	3.59 (2.47–5.21)^a^	182 (12.5)	1.82 (1.36-2.44)^a^
Lymphoma	51 (5.7)	6.69 (3.82–11.73)^a^	79 (5)	4.22 (2.72–6.55)^a^	38 (4.8)	3.21 (1.75–5.88)^a^	65 (4.5)	1.75 (1.06–2.91)^c^
Melanoma	13 (1.5)	N/E	30 (1.9)	0.13 (0.02–0.95)^a^	15 (1.9)	0.75 (0.22–2.58)	38 (2.6)	0.52 (0.19–1.48)
Myeloma	5 (0.6)	6.44 (1.16–35.86)^c^	18 (1.2)	1.42 (0.53–3.77)	6 (0.8)	0.80 (0.09–6.78)	22 (1.5)	0.56 (0.16–1.90)
Other	7 (0.8)	1.79 (0.35–9.22)	13 (0.8)	1.95 (0.6–6.31)	22 (2.8)	2.59 (1.06–6.34)^c^	38 (2.6)	1.28 (0.59–2.75)
Ovary					33 (4.2)	3.47 (1.75–6.91)^a^	67 (4.6)	1.59 (0.91–2.76)^a^
Pancreas	47 (5.3)	13.31 (6.79–26.1)^a^	66 (4.2)	6.86 (4.11–11.43)^a^	46 (5.8)	4.30 (2.47–7.5)^a^	75 (5.2)	2.92 (1.85–4.60)^a^
Prostate	150 (16.8)	0.79 (0.53–1.19)	336 (21.5)	0.51 (0.37–0.71)^a^				
Renal tract	78 (8.7)	2.16 (1.36–3.43)^a^	130 (8.3)	1.61 (1.1–2.35)^a^	47 (6.0)	1.85 (1.01–3.39)	76 (5.2)	1.26 (0.76–2.08)
Uterine					39 (5.0)	1.29 (0.61–2.7)^c^	63 (4.3)	0.84 (0.43–1.67)

Cancer stage	*n* (%)^d^	UWL HR (95% CI)	*n* (%)^d^	UWL HR (95% CI)	*n* (%)^d^	UWL HR (95% CI)	*n* (%)^d^	UWL HR (95% CI)
Stage I	11 (11.3)	0.73 (0.21–2.58)	21 (12.9)	0.53 (0.18–1.54)	23 (24.2)	1.31 (0.52–3.3)	39 (25.3)	0.61 (0.26–1.45)
Stage II	12 (12.4)	3.38 (1.3–8.82)^a^	27 (16.6)	1.39 (0.66–2.91)	12 (12.6)	1.31 (0.42–4.03)	25 (16.2)	0.86 (0.33–2.26)
Stage III	16 (16.5)	2.53 (1.12–5.69)^c^	25 (15.3)	2.11 (1.05–4.22)^c^	13 (13.7)	1.54 (0.54–4.37)	20 (13)	0.85 (0.32–2.27)
Stage IV	58 (59.8)	6.89 (4.22–11.24)^a^	90 (55.2)	3.79 (2.57–5.61)^a^	47 (49.5)	5.32 (3.06–9.24)^a^	70 (45.5)	2.93 (1.86–4.59)^a^
Early-stage	23 (23.7)	1.72 (0.83–3.55)	48 (29.4)	0.95 (0.52–1.73)	35 (36.8)	1.31 (0.64–2.68)	64 (41.6)	0.71 (0.37–1.34)
Late-stage	74 (76.3)	5.22 (3.46–7.88)^a^	115 (70.6)	3.26 (2.32–4.58)^a^	82 (86.3)	3.96 (2.47–6.34)^a^	90 (58.4)	2.25 (1.51–3.34)^a^

Each model is adjusted for age-group, smoking status, alcohol intake, ethnicity, deprivation, BMI-group and family history of cancer and stratified by comorbidity.

*N/E* not estimable.

^a^*p* ≤ 0.01.

^b^SRT ≤ 0.05.

^c^*p* ≤ 0.05.

^d^Percentage for cancer stage represents percentage of staged cancers.

#### Sensitivity analyses

We conducted four sensitivity analyses. Firstly, we assessed further how the HR changed over time by fitting flexible parametric Royston-Parmar (RP) models for cancer overall, adjusted for all covariates, using restricted cubic splines with 4 degrees of freedom.^23^ Secondly, we calculated HRs including a matching variable in the Cox models to assess the effect of including a matching variable on HR precision, because we wanted to assess whether ignoring the matching variable led to significant bias.^24^ Thirdly, to identify thresholds that could inform investigative action, we calculated the predicted HR for cancer in the next 3 months for a person with unexpected weight loss based on their age and gender by substituting age-group with age as a continuous variable in the 0–3 m Cox models, centring age at 60 years, including interactions between unexpected weight loss, age and gender, and calculating marginal effects at representative values of age.^25,26^ These interactions were chosen based on previous literature reporting a different effect of unexpected weight loss by gender and age.^15^ Finally, to assess the validity of our results, we fitted multinomial logistic regression models to assess whether the association between unexpected weight loss and a diagnosis of cancer in the next six months was similar to the Cox models for each cancer site in the primary analysis.

## Results

### Summary of cohort

In all, 63,973 of 4,832,140 (1.3%) patients had at least one record of unexpected weight loss in the eligible population (Fig. 1) (1.5% prior to exclusions). Table 1 details how patients with unexpected weight loss compared to comparators. In all, 1375 of 63,973 patients (2.2%) with unexpected weight loss and 8,285 of 266,471 patients (3.1%) without unexpected weight loss were diagnosed with cancer over 2-years of follow-up. The time to diagnosis was significantly shorter in patients with unexpected weight loss (median 80 days (IQR 26–290)), with a positive skew (mean 181 days (standard deviation 205), compared to patients without (median 353 (IQR 181-541), *p* < 0.01) (Supplementary information 2).

### Risk of cancer diagnosis following presentation with unexpected weight loss

**Fig. 2:**
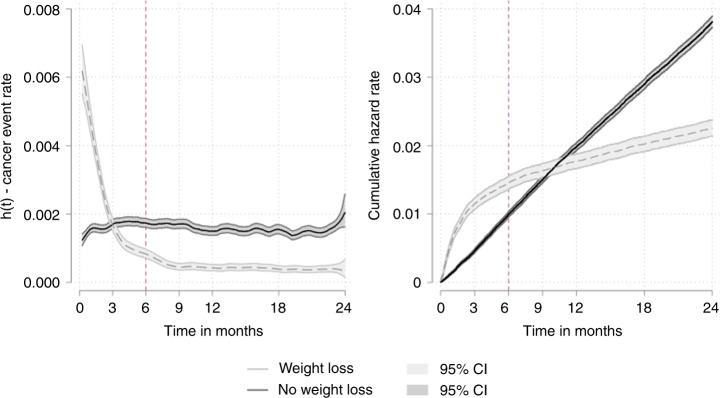
Risk of cancer diagnosis over time. Univariate smoothed hazard rate and cumulative hazard of cancer diagnosis by unexpected weight loss status.

Over the 2 years, the risk of a cancer diagnosis was higher in patients with unexpected weight loss than controls in the three months after the index consultation, but thereafter, the risk of cancer was lower than controls (Fig. 2—left panel, Supplementary information 3). The same pattern was observed after adjustment for potential confounders and after taking into account matching in the first and second sensitivity analyses (Table 2, Supplementary information 4). Expressed as the cumulative hazard of cancer, 1.2% of patients with unexpected weight loss and 0.5% without unexpected weight loss had been diagnosed with cancer in the first three months after the index consultation, a difference that narrowed to 1.6% and 1.5% by 9 months and then inverted to 1.8% and 2.0% by 12 months (Fig. 2—right panel).

### Gender and age-group

**Fig. 3:**
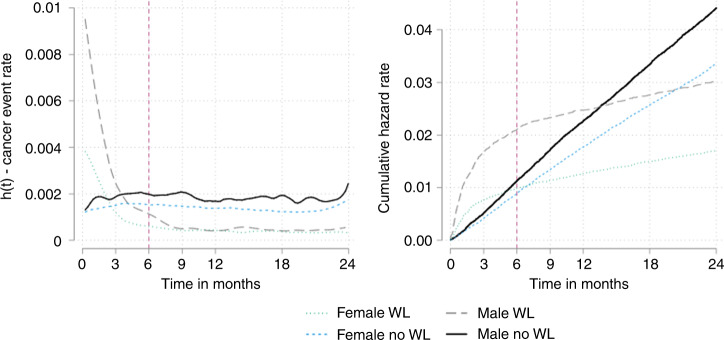
Risk of cancer over time by gender. Univariate smoothed hazard rate and cumulative hazard of cancer diagnosis by gender in patients with and without unexpected weight loss. In men, 441 cancers (1.7%) were diagnosed in 25,551 men with unexpected weight loss in the first 3 months rising to 548 (2.1%) cases over the first 6 months compared with 575 (0.5%) in 10,7706 men without unexpected weight loss in the first 3 months rising to 1215 (1.1%) by 6 months. In women, 282 cancers (0.8%) were diagnosed in 36,057 women with unexpected weight loss in the first 3 months rising to 360 (1.0%) over 6 months compared with 630 (0.4%) in 145,203 women without unexpected weight loss over 3 months rising to 1305 (0.9%) over 6 months.

**Fig. 4:**
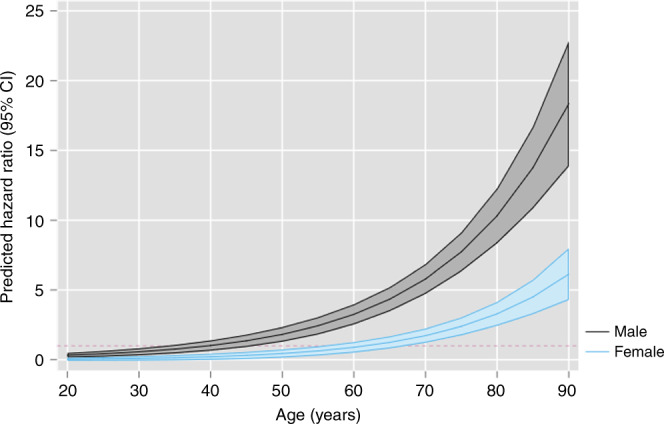
Predicted hazard ratios by age and gender. Population level predictions of the hazard ratio for cancer in the next three months for people with unexpected weight loss by age and gender using marginal effects at representative values.

Overall, the time to diagnosis was significantly shorter in men with unexpected weight loss (median 67 days (IQR 22–212)) than women (median 111 days (IQR 33–358)) (Supplementary information 5). In the first 3 months men with unexpected weight loss had more than a 3-fold likelihood of being diagnosed with cancer compared to comparators (HR 3.28 (2.88–3.73)) in the Cox models, whereas for women it was less (1.85 (1.6–2.14)) (Fig. 3, Table 2). The same difference between sexes was seen across the first 6 months (Fig. 3, Table 2). In the third sensitivity analysis, there were statistically significant interactions between unexpected weight loss and gender (*p* < 0.01), gender and age (*p* < 0.01), and unexpected weight loss, age and gender (*p* < 0.01). Unexpected weight loss was associated with a decreased risk of cancer in men under the age of 35 years compared to controls (Fig. 4, Supplementary information 6) Between 35 and 50 years, there was no evidence of an association in men, and above 50 years unexpected weight loss was associated with an increasing risk of cancer (1.82 (1.30–2.34) at 50 years). For women, the risk of cancer was not increased until 70 years old (1.73 (1.22–2.24)) (Fig. 4, Supplementary Information 6)

### Cancer site and stage

**Fig. 5:**
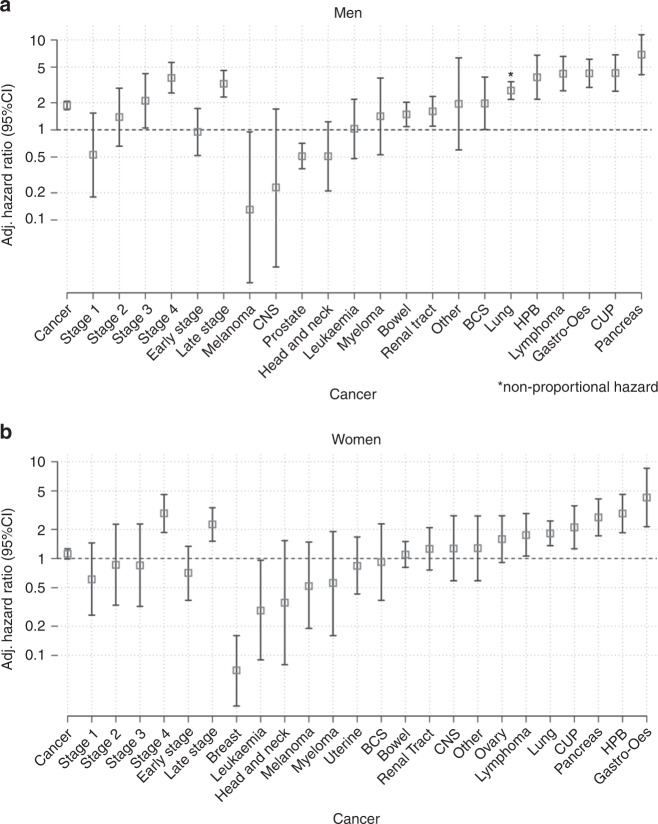
Risk of cancer by site and stage. Adjusted hazard ratios for cancer in people with unexpected weight loss in men (5a) and women (5b) in the 6 months following presentation derived using cox regression adjusted for all covariates.

Ordering the cancer sites by strength of association from the Cox modelling showed a similar pattern when considering HRs for the first three and first 6 months (Fig. 5, Table 2, Supplementary Information 7) and risk ratios for the first 6 months in the final sensitivity analysis (Supplementary Information 8).

Unexpected weight loss was associated with an increased likelihood of pancreatic cancer, cancer of unknown primary, gastro-oesophageal, lymphoma, HPB, lung, bowel and renal tract compared with controls. Unexpected weight loss was associated with a decreased likelihood of breast and prostate cancer than controls (Fig. 5, Table 2). Bone connective and soft tissue cancers were significantly increased in men over 3 months and 6 months. Myeloma in men and ovarian in women were significantly increased over 3 months in patients with unexpected weight loss but not over 6 months (Fig. 5, Table 2). Bowel and renal tract cancer were not significantly increased at 6 months for women with unexpected weight loss but were at 3 months (Fig. 5, Table 2).

**Fig. 6:**
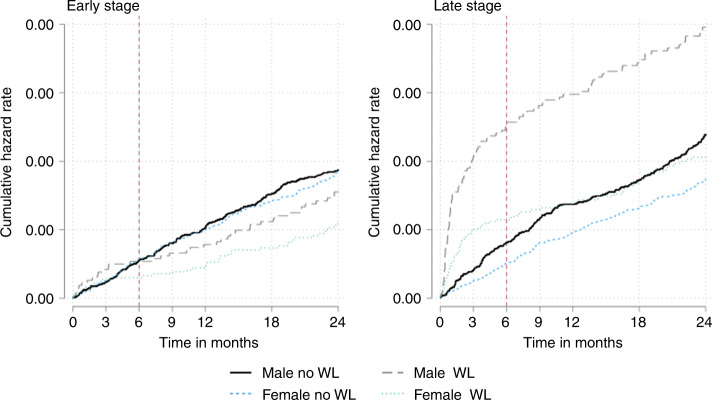
Risk of cancer over time by cancer stage. Univariate smoothed hazard rate and cumulative hazard by cancer stage in patients with and without unexpected weight loss. In men, 54 cancers (0.2%) were diagnosed with late-stage cancer in 25,551 men with unexpected weight loss in the first 3 months rising to 66 (0.3%) cases over the first 6 months compared with 46 (0.04%) of 10,7706 men without unexpected weight loss in the first 3 months rising to 88 (0.08%) by 6 months. In women, 37 late-stage cancers (0.1%) were diagnosed in 36,057 women with unexpected weight loss in the first 3 months rising to 52 (0.1%) over 6 months compared with 38 (0.02%) in 145,203 women without unexpected weight loss over 3 months rising to 75 (0.05%) over 6 months.

For both genders, unexpected weight loss was associated with increased late-stage cancer diagnoses (Fig. 6). This was most often stage IV cancer but there was also an increased likelihood of stage III in men (Table 2). An increased likelihood of stage II cancer was observed for men (3.38 (1.3–8.82)) but there was no difference when earlier-stage cancers were combined.

## Discussion

### Summary of findings

Patients for whom their GP records unexpected weight loss are at increased risk of the following cancers within the next three months: pancreatic, cancer of unknown primary, gastro-oesophageal, lymphoma, hepatobiliary, lung, bowel and renal tract. The association is greatest in men aged ≥50 years and in women aged ≥70 years. Conversely, breast cancer and prostate cancer are less likely with unexpected weight loss than comparators. Most cancers associated with unexpected weight loss are diagnosed at a late-stage, but there was also evidence for an association between unexpected weight loss and the diagnosis of stage II and stage III cancers. After the initial three-month period, the risk of a cancer diagnosis dropped below the risk of cancer in patients without unexpected weight loss, and this reduction is sustained for the following 21 months.

### Strengths and limitations

To our knowledge this is the largest study to examine the association between unexpected weight loss and subsequent cancer diagnosis in primary care. By using a matched-cohort design and time-to-event methods we have identified the period of time with the greatest likelihood of a cancer diagnosis. We adjusted for potential confounders to establish the independent association between weight loss and cancer. By using cancer registry linked data we have been able to investigate the association between unexpected weight loss and cancer stage, however, <15% of cancers had complete staging data available.

We took care to ensure that the cohort with unexpected weight loss was accurately defined. Firstly, we relied on coded unexpected weight loss entries, as UK GPs do not weigh patients frequently enough to allow reliable identification of weight loss in the EHR.^20,27,28^ Secondly, our internal validation study identified the codes which most consistently defined weight loss.^4,5,19^ Thirdly, we excluded patients with a past history of cancer as they are at higher risk of cancer than the primary care population,^29^ unlike several previous studies.^30,31^ Including patients with past cancer would bias the study and lead to inflated estimates of association. Other studies have adjusted for previous cancer, though without considering how long ago the cancer was diagnosed.^32,33^ Fourthly, we excluded patients with evidence of intentional weight loss with medication or surgery. Finally, when related to a common disease state, unexpected weight loss usually represents a deterioration or complication, such as end-stage COPD or wasting in dementia.^34^ The prevalence of these conditions is potentially high, and it is challenging to identify severity of disease in EHRs. We therefore adjusted for comorbidity in our multivariable analyses.

Most previous studies have used a 1–2 year pre-diagnostic period to examine the association between unexpected weight loss and cancer diagnosis.^16^ A common justification is ‘a 2-year period was used, since this represents the period of time during which existing cancers are likely to become clinically manifest’.^32,33^ This does not allow the predictive value of a given symptom to vary during different periods before diagnosis. We show that the probability of cancer is initially much higher in patients with unexpected weight loss, once it is recorded, after which it falls below the probability in patients without unexpected weight loss. This non-proportional hazard highlights the mechanism that could lead to inaccurate estimates of association between symptoms and cancer diagnoses. For example, the probability of cancer could be underestimated with too long a follow-up period: initially high probabilities are counterbalanced by later lower probabilities attenuating the hazard ratio to the null. Similarly, predictive values could be overestimated as cancers occurring after the ‘at-risk’ period would be incorrectly classified as true positives. Restricting our analysis to the period of greatest probability resulted in low event numbers for some cancer sites and uncertainty of the estimates of association. Creating larger cancer groups to overcome this would have countered our intent to define clinically sensible groups of cancers that are diagnosed in a common pathway. To assess whether the results were robust, we examined different statistical models and the strengths of association between cancer site and stage over the first 3 and first 6 months after presenting with unexpected weight loss and found similar results whichever analysis was used.

### Findings in context

A systematic review found evidence that unexpected weight loss was associated with 10 different cancers.^15^ Since then, English and Swedish case–control studies have reported that weight loss is associated with acute and chronic leukaemia and non-metastatic colorectal cancer.^35,36^ For some cancer sites there have been no studies on the association with unexpected weight loss in primary care. Our analysis is the first to focus on the association between unexpected weight loss and cancer across all sites.

Few studies have reported the association between unexpected weight loss and cancer stage in primary care.^15^ A recent cross-sectional study using data from the English National Cancer Diagnosis Audit (NCDA) of reported that 49% of 584 cancers associated with unexpected weight loss were diagnosed at stage IV.^37^ In our study, 66–70% of staged cancers were diagnosed at stage IV, depending on gender and the period of follow-up. These differences are likely to relate to the method of data collection: the NCDA relied on retrospective review of coded and uncoded (free text) EHR data whereas our study relied on coded EHR data only. A previous study investigating ovarian cancer diagnosis showed that weight loss was reported more frequently when data were collected by questionnaire or telephone interview compared with using only coded EHR data.^38^

Our finding that breast cancer is negatively associated with the recording of unexpected weight loss has not been reported before. A case–control study excluded unexpected weight loss as it was present in <1% of patients, reflecting that breast cancer much more commonly follows other symptoms in primary care.^39^ A negative association means that breast cancer may still be diagnosed following unexpected weight loss, but this is less likely than in patients without unexpected weight loss. Our finding that prostate cancer is negatively associated with unexpected weight loss conflicts with two previous primary care studies reporting positive associations.^32,40^ This difference could be a consequence of the longer period of follow-up used without consideration of non-proportional hazards. Our estimate of association between unexpected weight loss and myeloma was imprecise as there were few with myeloma, but a previous case–control study found an increased risk of myeloma in the 3 months following presenting with unexpected weight loss.^41^

The literature links unexpected weight loss with long delays in the interval between the symptom onset and the final cancer diagnosis (the diagnostic interval).^6^ Our analysis shows that patients are diagnosed with cancer within three months of their GP recording it (the health system interval). Walter et al. reported unexpected weight loss as the symptom associated with the longest interval between symptom onset and GP presentation (the patient interval) in patients later diagnosed with pancreatic cancer.^10^ Further research is required to understand how patients experience unexpected weight loss prior to a diagnosis of cancer to inform strategies to promote earlier GP attendance.

### Implications for research and practice

Our findings have important implications for future research. NICE recommend urgent cancer investigation for patients with clinical features with a positive predictive value ≥3%. We demonstrate that predictive values derived from observational studies may be incorrect if a follow-up period longer (or shorter) than the symptoms’ ‘at-risk’ period is used. Studies used to inform policy should therefore report estimates of association for the period of increased risk, which will likely vary by symptom and cancer site.

Previous studies suggest that health-care systems focussed on investigating individual cancer sites based on alarm symptoms might disadvantage patients with non-specific symptoms.^12^ A Danish study showed that 6% of patients who received a negative result after single cancer site investigation were re-referred within 6 months to another, 4.4% of whom are diagnosed with cancer.^42^ Our results show that unexpected weight loss is associated with several cancer sites and so a broader investigative strategy may be better suited to these patients than one focussed on ruling-out a single cancer site. Focus in Northern Europe has shifted to the role of multidisciplinary diagnostic centres (MDCs) to achieve this.^12,14,43,44^ Emerging evidence from MDC pilot sites in the UK show that unexpected weight loss is the most common reason for referral.^14,44^ Insights gained from the ongoing evaluation of MDCs may inform the optimal diagnostic process for patients referred with unexpected weight loss.

As unexpected weight loss is a symptom of a wide range of benign and serious conditions related to almost any bodily system, cancer must not be considered in isolation.^34,45^ Evidence from MDCs shows that serious disease, other than cancer, is diagnosed in up to one third of patients referred.^11^ Most patients presenting to primary care with unexpected weight loss will not have cancer or serious disease and based on this study only 0.2% of patients under 60 years old will have cancer. In many countries, efforts are underway to reduce unnecessary medical intervention that does not add value for patients and may even cause harm.^46^ In the US, the Institute of Medicine estimates that $105 billion could be saved annually by reducing unnecessary intervention by 50%.^47^ In the UK, increasing diagnostic activity in the UK related to ‘alarm’ symptoms with a PPV ≥ 3% is already stretching diagnostic capacity.^48,49^ In this study the approximate PPVs for a cancer diagnosis within 3 months in men aged ≥50 years and women aged ≥70 years with unexpected weight loss were 2.4% and 1.5%, respectively, rising to 3.0% and 1.9% over a 6-month period. Research is urgently required to identify patients with unexpected weight loss at greatest risk of cancer and other serious diseases to inform evidence-based guidelines by using additional risk factors, clinical features, and primary care tests. These tests would have to be easily accessible to GPs and reported quickly to avoid delays in referral to the appropriate speciality.

For patients with unexpected weight loss not initially investigated for possible cancer, or in those with ongoing unexpected weight loss for whom initial investigation does not identify malignancy, primary care clinicians could employ a policy of watchful waiting over the subsequent 3 months when the risk of a cancer being detected is greatest. During this time, repeat evaluation may consider the necessity of further investigation and referral.

## Conclusion

Presentation and recording of a first presentation of unexpected weight loss is uncommon in primary care (1.5% patients during the 14-year study period). However, unexpected weight loss may be a presenting feature of cancer in primary care that, when recorded in older people, warrants early investigation across a broad range of cancer sites. Future analyses must establish the role of risk factors and accompanying symptoms, signs and simple primary care tests that could safely be used to select patients with unexpected weight loss for further intensive cancer investigation.

## Supplementary information

Supplementary information

## Data Availability

This study is based on CPRD data and is subject to a full licence agreement which does not permit data sharing outside of the research team.
