# Supplementary material for: The association between unexpected weight loss and cancer diagnosis in primary care: a matched cohort analysis of 65,000 presentations
Source: Br J Cancer. 2020 Apr 15;122(12):1848–56. doi: 10.1038/s41416-020-0829-3 (PMC7283307; doi:10.1038/s41416-020-0829-3)
Supplement: Supplementary file 1 — Supplementary information [file 41416_2020_829_MOESM1_ESM.docx]

**Supplementary information to accompany manuscript**:

“The association between unexpected weight loss and cancer diagnosis in primary care: a matched cohort analysis of 65,000 presentations over 14 years” by Nicholson BD et al.

**Supplementary information 1**: Validated Clinical Practice Research Datalink (CPRD) codes used to define unexpected weight loss.

| medcode | Read term | n (%) |
| --- | --- | --- |
| 654 | Weight Decreasing | 6439 (10.07) |
| 3647 | [D]Abnormal loss of weight | 15738 (24.6) |
| 4663 | Abnormal weight loss | 2551 (3.99) |
| 5812 | Abnormal weight loss - symptom | 31562 (49.34) |
| 12398 | Complaining of weight loss | 4809 (7.52) |
| 22005 | O/E - cachexic | 45 (0.07) |
| 24068 | [D]Cachexia | 249 (0.39) |
| 37937 | Weight loss from baseline weight | 2537 (3.97) |
| 102563 | Unintentional weight loss | 43 (0.07) |
| Total | | **63973 (100)** |

**Supplementary information 2:** Distribution and diagnostic intervals for cancers diagnosed in the two years following the index date stratified by UWL status.

| Cancer | All | UWL | | | No UWL | | | K-Sample Test |
| --- | --- | --- | --- | --- | --- | --- | --- | --- |
|  |  | **Cancers** | **Diagnostic Interval (days)** | | **Cancers** | **Diagnostic Interval (days)** | |  |
|  | *n (% of All)* | *n (% of UWL)* | *Median (IQR)* | *Mean (SD)* | *n (% of no UWL)* | *Median (IQR)* | *Mean (SD)* | *p-value* |
| Overall | 9660 (2.92) | 1375 (2.15) | 80 (26-290) | 181 (205) | 8285 (3.11) | 353 (181-541) | 361 (209) | <0.01 |
|  |  |  |  |  |  |  |  |  |
| Stage 1 | 260 (0.08) | 41 (0.06) | 404 (194-551) | 372 (224) | 219 (0.08) | 395 (201-525) | 375 (202) | 1.00 |
| Stage 2 | 227 (0.07) | 35 (0.05) | 314 (24-607) | 314 (270) | 192 (0.07) | 463 (240-610) | 427 (213) | 0.08 |
| Stage 3 | 180 (0.05) | 34 (0.05) | 166 (17-341) | 210 (213) | 146 (0.05) | 432 (214-652) | 418 (231) | <0.01 |
| Stage 4 | 436 (0.13) | 139 (0.22) | 76 (25-416) | 202 (230) | 297 (0.11) | 404 (203-580) | 391 (218) | <0.01 |
|  |  |  |  |  |  |  |  |  |
| Lung | 1417 (0.43) | 309 (0.48) | 63 (24-210) | 155 (187) | 1108 (0.42) | 362 (190-541) | 368 (207) | <0.01 |
| Bowel | 1394 (0.42) | 180 (0.28) | 89 (30-311) | 190 (203) | 1214 (0.46) | 351 (188-535) | 358 (208) | <0.01 |
| Prostate | 1260 (0.38) | 76 (0.12) | 142 (42-438) | 247 (227) | 1184 (0.44) | 366 (184-552) | 370 (209) | <0.01 |
| Breast | 1183 (0.36) | 26 (0.04) | 426 (188-593) | 400 (237) | 1157 (0.43) | 341 (173-532) | 353 (209) | 0.16 |
| Renal Tract | 703 (0.21) | 96 (0.15) | 111 (30-277) | 177 (183) | 607 (0.23) | 346 (188-532) | 358 (207) | <0.01 |
| Gastro-Oesophageal | 562 (0.17) | 141 (0.22) | 42 (16-195) | 138 (189) | 421 (0.16) | 350 (196-533) | 361 (207) | <0.01 |
| Lymphoma | 407 (0.12) | 94 (0.15) | 80 (23-205) | 146 (166) | 313 (0.12) | 363 (187-533) | 368 (212) | <0.01 |
| Pancreatic | 348 (0.11) | 111 (0.17) | 56 (17-219) | 158 (203) | 237 (0.09) | 379 (179-556) | 372 (215) | <0.01 |
| Cancer of Unknown Primary | 325 (0.10) | 87 (0.14) | 58 (15-223) | 152 (200) | 238 (0.09) | 314 (157-514) | 339 (211) | <0.01 |
| Leukaemia | 280 (0.08) | 18 (0.03) | 141 (63-302) | 208 (200) | 262 (0.10) | 371 (189-584) | 376 (217) | 0.03 |
| Melanoma | 278 (0.08) | 10 (0.02) | 184 (68-612) | 299 (261) | 268 (0.10) | 386 (189-567) | 382 (211) | 0.75 |
| Ovary | 210 (0.06) | 32 (0.05) | 114 (44-540) | 257 (254) | 178 (0.07) | 342 (174-555) | 355 (213) | 0.18 |
| Head & Neck | 202 (0.06) | 19 (0.03) | 186 (106-557) | 297 (250) | 183 (0.07) | 329 (168-497) | 342 (206) | 0.63 |
| Uterine | 195 (0.06) | 20 (0.03) | 149 (42-528) | 273 (266) | 175 (0.07) | 335 (152-521) | 342 (215) | 0.49 |
| Hepatobiliary | 192 (0.06) | 55 (0.09) | 61 (20-161) | 166 (223) | 137 (0.05) | 345 (173-532) | 363 (210) | <0.01 |
| Bone Connective Soft Tissue | 190 (0.06) | 30 (0.05) | 95 (32-288) | 180 (202) | 160 (0.06) | 320 (136-526) | 336 (216) | 0.01 |
| Other | 182 (0.06) | 22 (0.03) | 149 (36-319) | 206 (209) | 160 (0.06) | 374 (205-566) | 371 (213) | 0.01 |
| Myeloma | 164 (0.05) | 24 (0.04) | 218 (127-449) | 268 (198) | 140 (0.05) | 379 (219-584) | 390 (207) | 0.05 |
| Central Nervous System | 155 (0.05) | 19 (0.03) | 133 (58-295) | 194 (162) | 136 (0.05) | 306 (130-534) | 328 (218) | 0.05 |

**Supplementary information 3:** The cumulative hazard (%) of cancer over a two year period in adults >18yrs in patients with and without unexpected weight loss (UWL). BCS = bone connective and soft tissue, CUP = cancer of unknown primary, CNS = central nervous system, Gastro-Oesoph = gastro-oesophageal.

| Cancer | UWL | Cumulative Hazard of cancer | | | | | | Wilcoxon test |
| --- | --- | --- | --- | --- | --- | --- | --- | --- |
|  | *(UWL / noUWL)* | **3 months** | **6 months** | **9 months** | **12 months** | **18 months** | **24 months** | **p-value** |
|  |  |  |  |  |  |  |  |  |
| Cancer | *UWL* | 1.15 (1.07-1.24) | 1.45 (1.36-1.55) | 1.64 (1.54-1.74) | 1.77 (1.67-1.88) | 2.02 (1.91-2.14) | 2.26 (2.14-2.38) | <0.001 |
|  | *noUWL* | 0.46 (0.44-0.49) | 1 (0.96-1.04) | 1.5 (1.46-1.55) | 1.98 (1.93-2.04) | 2.9 (2.83-2.97) | 3.81 (3.73-3.9) |  |
|  |  |  |  |  |  |  |  |  |
| Stage 1 | *UWL* | 0.01 (0.01-0.03) | 0.02 (0.01-0.03) | 0.02 (0.01-0.04) | 0.03 (0.02-0.05) | 0.05 (0.04-0.07) | 0.07 (0.05-0.09) | 0.02 |
|  | *noUWL* | 0.01 (0.01-0.02) | 0.03 (0.03-0.04) | 0.05 (0.04-0.06) | 0.06 (0.05-0.07) | 0.08 (0.07-0.09) | 0.1 (0.09-0.11) |  |
| Stage 2 | *UWL* | 0.02 (0.01-0.03) | 0.02 (0.01-0.04) | 0.03 (0.02-0.04) | 0.03 (0.02-0.05) | 0.04 (0.03-0.06) | 0.06 (0.04-0.08) | 0.04 |
|  | *noUWL* | 0.01 (0.01-0.01) | 0.02 (0.02-0.03) | 0.03 (0.03-0.04) | 0.04 (0.03-0.05) | 0.07 (0.06-0.08) | 0.09 (0.08-0.1) |  |
| Stage 3 | *UWL* | 0.02 (0.01-0.04) | 0.03 (0.02-0.05) | 0.04 (0.02-0.06) | 0.04 (0.03-0.06) | 0.05 (0.04-0.07) | 0.06 (0.04-0.08) | 0.69 |
|  | *noUWL* | 0.01 (0.01-0.02) | 0.02 (0.02-0.03) | 0.03 (0.03-0.04) | 0.04 (0.03-0.05) | 0.05 (0.04-0.06) | 0.07 (0.06-0.08) |  |
| Stage 4 | *UWL* | 0.12 (0.1-0.15) | 0.14 (0.12-0.18) | 0.16 (0.13-0.19) | 0.16 (0.13-0.2) | 0.19 (0.16-0.23) | 0.23 (0.19-0.27) | <0.001 |
|  | *noUWL* | 0.02 (0.02-0.03) | 0.04 (0.04-0.05) | 0.06 (0.05-0.08) | 0.08 (0.07-0.09) | 0.1 (0.09-0.12) | 0.14 (0.12-0.15) |  |
|  |  |  |  |  |  |  |  |  |
| Early Stage | *UWL* | 0.03 (0.02-0.05) | 0.04 (0.03-0.06) | 0.05 (0.03-0.07) | 0.06 (0.04-0.08) | 0.09 (0.07-0.12) | 0.13 (0.1-0.16) | <0.01 |
|  | *noUWL* | 0.02 (0.02-0.03) | 0.06 (0.05-0.07) | 0.08 (0.07-0.09) | 0.1 (0.09-0.11) | 0.15 (0.13-0.16) | 0.19 (0.17-0.21) |  |
| Late Stage | *UWL* | 0.14 (0.12-0.18) | 0.17 (0.14-0.21) | 0.19 (0.16-0.23) | 0.2 (0.17-0.24) | 0.24 (0.21-0.28) | 0.28 (0.25-0.33) | <0.001 |
|  | *noUWL* | 0.03 (0.03-0.04) | 0.06 (0.06-0.08) | 0.1 (0.08-0.11) | 0.11 (0.1-0.13) | 0.15 (0.13-0.17) | 0.2 (0.18-0.22) |  |
|  |  |  |  |  |  |  |  |  |
| BCS | *UWL* | 0.02 (0.01-0.04) | 0.03 (0.02-0.05) | 0.04 (0.02-0.05) | 0.04 (0.03-0.06) | 0.04 (0.03-0.06) | 0.05 (0.03-0.07) | 0.1597 |
|  | *noUWL* | 0.01 (0.01-0.01) | 0.02 (0.02-0.03) | 0.03 (0.02-0.04) | 0.04 (0.03-0.05) | 0.06 (0.05-0.07) | 0.07 (0.06-0.09) |  |
| Bowel | *UWL* | 0.14 (0.12-0.18) | 0.18 (0.15-0.22) | 0.21 (0.17-0.25) | 0.23 (0.2-0.27) | 0.26 (0.22-0.31) | 0.3 (0.26-0.34) | <0.001 |
|  | *noUWL* | 0.07 (0.06-0.08) | 0.14 (0.13-0.16) | 0.22 (0.21-0.24) | 0.3 (0.28-0.32) | 0.43 (0.41-0.46) | 0.56 (0.53-0.59) |  |
| Breast | *UWL* | 0.01 (0-0.02) | 0.01 (0-0.02) | 0.01 (0.01-0.02) | 0.02 (0.01-0.03) | 0.03 (0.02-0.05) | 0.04 (0.03-0.07) | <0.001 |
|  | *noUWL* | 0.06 (0.05-0.07) | 0.14 (0.13-0.16) | 0.21 (0.2-0.23) | 0.28 (0.26-0.3) | 0.41 (0.38-0.44) | 0.53 (0.5-0.56) |  |
| CUP | *UWL* | 0.08 (0.06-0.11) | 0.1 (0.08-0.13) | 0.11 (0.09-0.14) | 0.12 (0.09-0.15) | 0.13 (0.11-0.16) | 0.14 (0.11-0.17) | <0.01 |
|  | *noUWL* | 0.01 (0.01-0.02) | 0.03 (0.03-0.04) | 0.05 (0.04-0.06) | 0.06 (0.05-0.07) | 0.08 (0.07-0.1) | 0.11 (0.1-0.12) |  |
| CNS | *UWL* | 0.01 (0-0.02) | 0.02 (0.01-0.03) | 0.02 (0.01-0.04) | 0.03 (0.02-0.04) | 0.03 (0.02-0.05) | 0.03 (0.02-0.05) | 0.01 |
|  | *noUWL* | 0.01 (0.01-0.02) | 0.02 (0.01-0.03) | 0.03 (0.02-0.03) | 0.03 (0.03-0.04) | 0.05 (0.04-0.06) | 0.06 (0.05-0.07) |  |
| Gastro-Oesoph | *UWL* | 0.15 (0.12-0.18) | 0.16 (0.14-0.2) | 0.19 (0.15-0.22) | 0.2 (0.17-0.24) | 0.21 (0.18-0.25) | 0.23 (0.19-0.27) | <0.001 |
|  | *noUWL* | 0.02 (0.02-0.03) | 0.05 (0.04-0.06) | 0.08 (0.07-0.09) | 0.1 (0.09-0.11) | 0.15 (0.14-0.17) | 0.19 (0.18-0.21) |  |
| Head & Neck | *UWL* | 0.01 (0-0.02) | 0.01 (0.01-0.03) | 0.02 (0.01-0.03) | 0.02 (0.01-0.03) | 0.02 (0.01-0.04) | 0.03 (0.02-0.05) | <0.001 |
|  | *noUWL* | 0.01 (0.01-0.02) | 0.02 (0.02-0.03) | 0.03 (0.03-0.04) | 0.04 (0.04-0.05) | 0.07 (0.06-0.08) | 0.08 (0.07-0.1) |  |
| Hepatobiliary | *UWL* | 0.05 (0.04-0.07) | 0.07 (0.05-0.09) | 0.07 (0.05-0.09) | 0.07 (0.05-0.09) | 0.08 (0.06-0.1) | 0.09 (0.07-0.12) | <0.01 |
|  | *noUWL* | 0.01 (0.01-0.02) | 0.02 (0.01-0.03) | 0.02 (0.02-0.03) | 0.03 (0.03-0.04) | 0.05 (0.04-0.06) | 0.06 (0.05-0.07) |  |
| Leukaemia | *UWL* | 0.01 (0.01-0.02) | 0.02 (0.01-0.03) | 0.02 (0.01-0.04) | 0.02 (0.01-0.04) | 0.03 (0.02-0.05) | 0.03 (0.02-0.05) | <0.001 |
|  | *noUWL* | 0.01 (0.01-0.02) | 0.03 (0.02-0.04) | 0.05 (0.04-0.06) | 0.06 (0.05-0.07) | 0.09 (0.08-0.1) | 0.12 (0.11-0.14) |  |
| Lung | *UWL* | 0.29 (0.25-0.34) | 0.35 (0.31-0.4) | 0.39 (0.34-0.44) | 0.41 (0.36-0.46) | 0.47 (0.42-0.53) | 0.5 (0.45-0.56) | 0.02 |
|  | *noUWL* | 0.05 (0.05-0.06) | 0.13 (0.12-0.15) | 0.2 (0.18-0.22) | 0.26 (0.24-0.28) | 0.39 (0.37-0.42) | 0.51 (0.48-0.54) |  |
| Lymphoma | *UWL* | 0.08 (0.06-0.11) | 0.11 (0.09-0.14) | 0.12 (0.1-0.15) | 0.13 (0.11-0.16) | 0.14 (0.12-0.18) | 0.15 (0.13-0.19) | 0.05 |
|  | *noUWL* | 0.02 (0.01-0.02) | 0.04 (0.03-0.05) | 0.06 (0.05-0.07) | 0.08 (0.07-0.09) | 0.11 (0.1-0.12) | 0.14 (0.13-0.16) |  |
| Melanoma | *UWL* | 0 (0-0.01) | 0.01 (0-0.02) | 0.01 (0-0.02) | 0.01 (0-0.02) | 0.01 (0.01-0.02) | 0.02 (0.01-0.03) | <0.001 |
|  | *noUWL* | 0.01 (0.01-0.02) | 0.03 (0.02-0.04) | 0.04 (0.04-0.05) | 0.06 (0.05-0.07) | 0.09 (0.08-0.1) | 0.12 (0.11-0.14) |  |
| Myeloma | *UWL* | 0.01 (0-0.02) | 0.01 (0.01-0.03) | 0.02 (0.01-0.04) | 0.03 (0.02-0.04) | 0.04 (0.02-0.06) | 0.04 (0.03-0.06) | 0.06 |
|  | *noUWL* | 0 (0-0.01) | 0.01 (0.01-0.02) | 0.02 (0.02-0.03) | 0.03 (0.02-0.04) | 0.05 (0.04-0.06) | 0.07 (0.06-0.08) |  |
| Other | *UWL* | 0.02 (0.01-0.03) | 0.02 (0.01-0.04) | 0.02 (0.01-0.04) | 0.03 (0.02-0.05) | 0.03 (0.02-0.05) | 0.04 (0.02-0.06) | 0.01 |
|  | *noUWL* | 0.01 (0-0.01) | 0.02 (0.01-0.02) | 0.03 (0.02-0.04) | 0.04 (0.03-0.05) | 0.05 (0.04-0.06) | 0.07 (0.06-0.09) |  |
| Ovary | *UWL* | 0.02 (0.01-0.04) | 0.03 (0.02-0.05) | 0.03 (0.02-0.05) | 0.04 (0.02-0.05) | 0.04 (0.03-0.06) | 0.05 (0.04-0.08) | 0.06 |
|  | *noUWL* | 0.01 (0.01-0.01) | 0.02 (0.02-0.03) | 0.04 (0.03-0.04) | 0.04 (0.04-0.05) | 0.06 (0.05-0.07) | 0.08 (0.07-0.1) |  |
| Pancreatic | *UWL* | 0.1 (0.08-0.13) | 0.13 (0.1-0.16) | 0.14 (0.12-0.18) | 0.15 (0.12-0.18) | 0.16 (0.13-0.2) | 0.18 (0.15-0.22) | <0.001 |
|  | *noUWL* | 0.02 (0.01-0.02) | 0.03 (0.02-0.04) | 0.04 (0.04-0.05) | 0.05 (0.05-0.06) | 0.08 (0.07-0.09) | 0.11 (0.1-0.12) |  |
| Prostate | *UWL* | 0.04 (0.03-0.06) | 0.07 (0.05-0.09) | 0.07 (0.06-0.1) | 0.08 (0.06-0.11) | 0.11 (0.08-0.14) | 0.13 (0.1-0.16) | <0.001 |
|  | *noUWL* | 0.06 (0.05-0.07) | 0.14 (0.13-0.15) | 0.2 (0.19-0.22) | 0.28 (0.26-0.3) | 0.41 (0.38-0.44) | 0.55 (0.52-0.58) |  |
| Renal Tract | *UWL* | 0.07 (0.05-0.1) | 0.1 (0.08-0.13) | 0.12 (0.09-0.15) | 0.12 (0.1-0.16) | 0.15 (0.12-0.18) | 0.16 (0.13-0.19) | <0.001 |
|  | *noUWL* | 0.04 (0.03-0.04) | 0.07 (0.06-0.08) | 0.11 (0.1-0.12) | 0.15 (0.13-0.16) | 0.21 (0.19-0.23) | 0.28 (0.26-0.3) |  |
| Uterine | *UWL* | 0.01 (0.01-0.03) | 0.02 (0.01-0.03) | 0.02 (0.01-0.03) | 0.02 (0.01-0.03) | 0.02 (0.01-0.04) | 0.03 (0.02-0.05) | <0.001 |
|  | *noUWL* | 0.01 (0.01-0.02) | 0.03 (0.02-0.03) | 0.04 (0.03-0.04) | 0.04 (0.04-0.05) | 0.06 (0.05-0.07) | 0.08 (0.07-0.09) |  |

**Supplementary information 4:** Royston-Parmar flexible parametric survival models showing the dynamic hazard ration for cancer in patients with unexpected weight loss (UWL) by age and gender. Models are adjusted for all covariates.


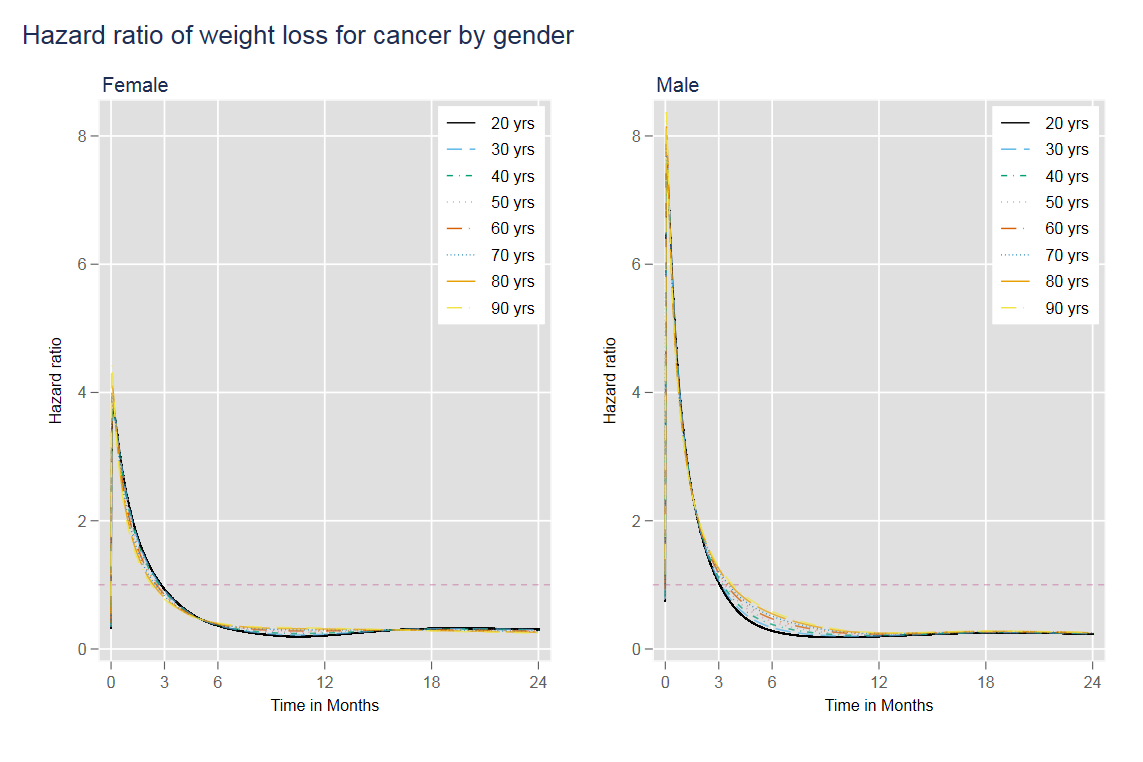


**Supplementary information 5**: Distribution and diagnostic intervals for cancers diagnosed in the two years following unexpected weight loss (UWL) in men and women.

| Cancer | UWL | Men | | | | Women | | | | K-Sample Test |
| --- | --- | --- | --- | --- | --- | --- | --- | --- | --- | --- |
|  |  | **Cancers** | **Diagnostic Interval** | | | **Cancers** | **Diagnostic Interval** | | |  |
|  | *N (%)* | *n (%)* | *Median (IQR)* | *Mean (SD)* | *Skew* | *n (%)* | *Median (IQR)* | *Mean (SD)* | *Skew* | *p-value* |
| Overall | 1375 (2.15) | 769 (2.87) | 67 (22-212) | 159 (195) | 1.44 | 606 (1.63) | 111 (33-358) | 208 (215) | 0.93 | <0.01 |
|  |  |  |  |  |  |  |  |  |  |  |
| Stage 1 | 41 (0.06) | 18 (0.07) | 430.5 (220-551) | 392 (226) | -0.11 | 23 (0.06) | 370 (78-566) | 356 (227) | -0.09 | 0.47 |
| Stage 2 | 35 (0.05) | 20 (0.07) | 182.5 (28.5-536) | 271 (255) | 0.35 | 15 (0.04) | 427 (24-675) | 372 (288) | -0.11 | 0.88 |
| Stage 3 | 34 (0.05) | 19 (0.07) | 82 (14-281) | 180 (229) | 1.28 | 15 (0.04) | 219 (54-369) | 248 (190) | 0.36 | 0.17 |
| Stage 4 | 139 (0.22) | 81 (0.3) | 74 (25-328) | 188 (219) | 1.11 | 58 (0.16) | 80 (26-500) | 221 (246) | 0.75 | 0.92 |
|  |  |  |  |  |  |  |  |  |  |  |
| Lung | 309 (0.48) | 196 (0.73) | 59.5 (20.5-183) | 143 (181) | 1.57 | 113 (0.3) | 87 (25-266) | 176 (197) | 1.11 | 0.32 |
| Bowel | 180 (0.28) | 91 (0.34) | 76 (29-305) | 191 (213) | 1.11 | 89 (0.24) | 104 (32-319) | 189 (194) | 1.08 | 0.37 |
| Gastro-Oesophageal | 141 (0.22) | 86 (0.32) | 37 (13-169) | 119 (176) | 1.99 | 55 (0.15) | 52 (18-245) | 168 (206) | 1.37 | 0.15 |
| Pancreatic | 111 (0.17) | 58 (0.22) | 50.5 (11-151) | 133 (196) | 1.88 | 53 (0.14) | 90 (29-269) | 185 (208) | 1.14 | 0.40 |
| Renal Tract | 96 (0.15) | 56 (0.21) | 71.5 (29-196.5) | 155 (176) | 1.46 | 40 (0.21) | 163.5 (53-334) | 208 (190) | 0.86 | 0.23 |
| Lymphoma | 94 (0.15) | 55 (0.21) | 58 (20-149) | 109 (131) | 1.91 | 39 (0.1) | 92 (52-344) | 197 (197) | 0.9 | 0.40 |
| Cancer of Unknown Primary | 87 (0.14) | 48 (0.18) | 53 (9.5-124) | 119 (172) | 1.94 | 39 (0.1) | 68 (15-345) | 193 (227) | 1.06 | 0.60 |
| Hepatobiliary | 55 (0.09) | 33 (0.12) | 61 (18-134) | 172 (230) | 1.4 | 22 (0.06) | 56.5 (21-161) | 158 (217) | 1.49 | 0.87 |
| Bone Connective Soft Tissue | 30 (0.05) | 15 (0.06) | 37 (31-129) | 106 (162) | 2.82 | 15 (0.04) | 209 (42-390) | 253 (216) | 0.43 | 0.11 |
| Myeloma | 24 (0.04) | 11 (0.04) | 136 (33-298) | 176 (159) | 0.98 | 13 (0.03) | 351 (199-489) | 346 (199) | 0.13 | 0.10 |
| Other | 22 (0.03) | 4 (0.01) | 108.5 (49-148.5) | 99 (62) | -0.34 | 18 (0.05) | 197.5 (36-348) | 230 (223) | 0.75 | 0.27 |
| Head & Neck | 19 (0.03) | 14 (0.05) | 184.5 (106-427) | 275 (251) | 0.65 | 5 (0.01) | 448 (120-557) | 356 (265) | -0.29 | 0.89 |
| Central Nervous System | 19 (0.03) | 8 (0.03) | 268.5 (207.5-473) | 302 (176) | -0.25 | 11 (0.03) | 92 (39-133) | 116 (98) | 1.19 | 0.01 |
| Leukaemia | 18 (0.03) | 11 (0.04) | 82 (43-151) | 170 (221) | 1.79 | 7 (0.02) | 258 (146-436) | 268 (158) | 0.38 | 0.05 |
| Melanoma | 10 (0.02) | 4 (0.01) | 409 (155.5-613.5) | 385 (268) | -0.07 | 6 (0.02) | 115 (58-404) | 242 (264) | 1.02 | 0.52 |

**Supplementary information 6:** Predicted hazard ratios for a cancer diagnosis within 3 months by age and gender for patients with unexpected weight loss (UWL) using marginal effects at representative values.

| **Age (yrs)** | **men** | **women** |
| --- | --- | --- |
|  | **HR (95% CI)** | **HR (95% CI)** |
| **20** | 0.32 (0.15-0.48) | 0.04 (-0.07-0.14) |
| **25** | 0.43 (0.22-0.63) | 0.06 (-0.07-0.19) |
| **30** | 0.57 (0.32-0.82) | 0.09 (-0.06-0.25) |
| **35** | 0.76 (0.46-1.07) | 0.14 (-0.04-0.32) |
| **40** | 1.02 (0.65-1.38) | 0.21 (0.00-0.43) |
| **45** | 1.36 (0.92-1.80) | 0.31 (0.06-0.56) |
| **50** | **1.82 (1.30-2.34)** | 0.44 (0.16-0.73) |
| **55** | 2.43 (1.82-3.04) | 0.63 (0.30-0.96) |
| **60** | 3.25 (2.53-3.97) | 0.89 (0.51-1.27) |
| **65** | 4.34 (3.48-5.19) | 1.25 (0.82-1.68) |
| **70** | 5.79 (4.73-6.85) | **1.73 (1.22-2.24)** |
| **75** | 7.73 (6.33-9.12) | 2.39 (1.76-3.02) |
| **80** | 10.31 (8.35-12.28) | 3.28 (2.43-4.14) |
| **85** | 13.76 (10.83-16.68) | 4.50 (3.26-5.73) |
| **90** | 18.35 (13.87-22.83) | 6.14 (4.28-7.99) |

**Supplementary information 7:** Ranked hazard ratios for a cancer diagnosis the 3-month period following unexpected weight loss by cancer site and stage by gender.

**Supplementary information 7a - men**

*
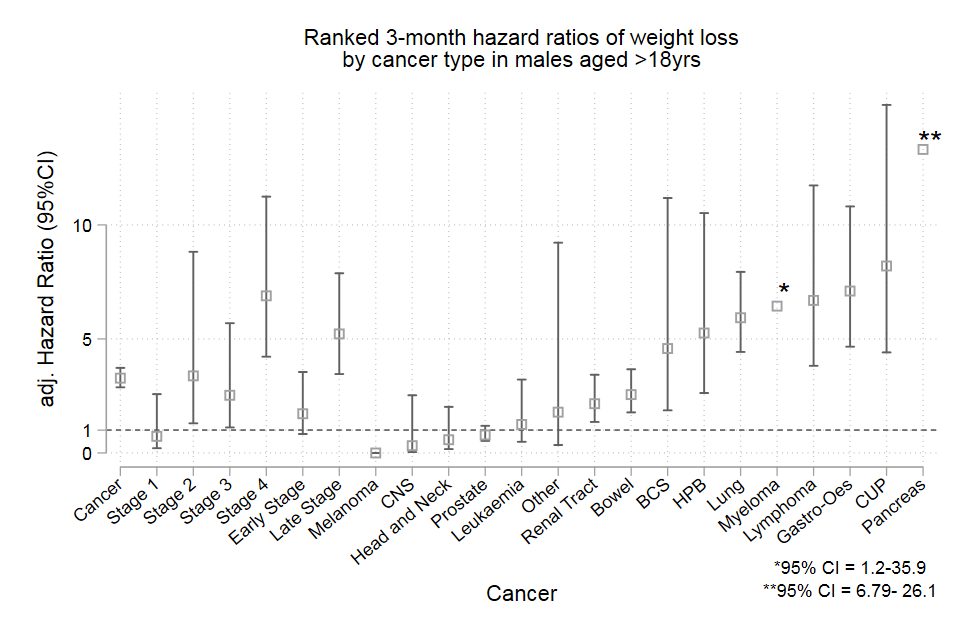
*

**Supplementary information 7b - women**

***.***

*
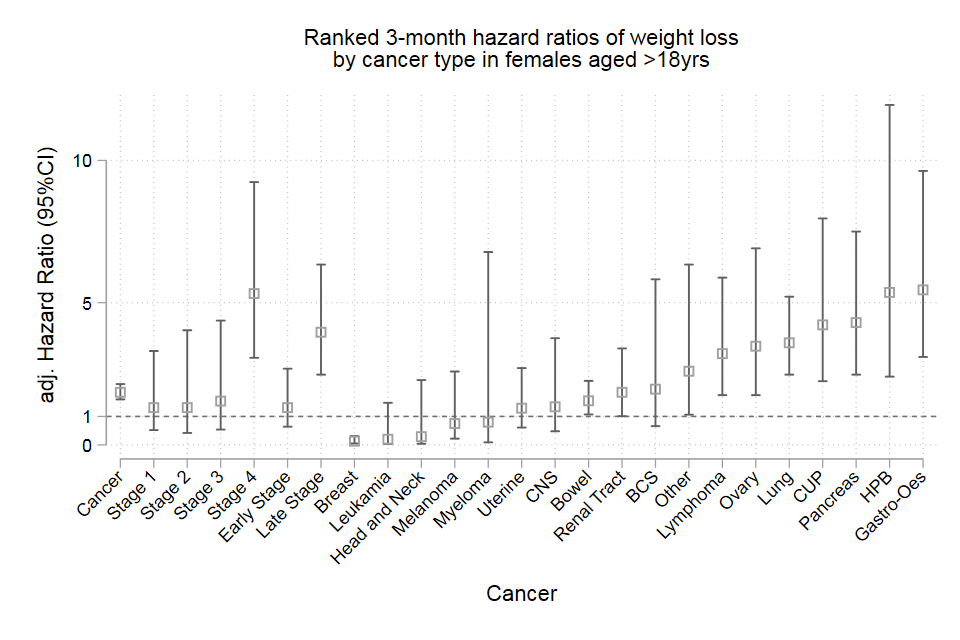
*

**Supplementary information 8:** Adjusted risk ratios for cancer in patients with unexpected weight loss (UWL) in men and women in the 6 months following presentation derived using multinomial logistic regression adjusted for all covariates.

**Supplementary information 8a.** Table of values.

| Cancer Site | Men | | Women | |
| --- | --- | --- | --- | --- |
|  | **adj RR (95% CI)** | **p-value** | **adj RR (95% CI)** | **p-value** |
|  |  |  |  |  |
| Cancer | 2.3 (2-2.5) | <0.001 | 1.4 (1.2-1.6) | <0.001 |
|  |  |  |  |  |
| Stage 1 | 1 (0.3-3.2) | 0.989 | 0.9 (0.4-2.3) | 0.869 |
| Stage 2 | 2.1 (0.9-4.9) | 0.010 | 1.8 (0.6-4.9) | 0.285 |
| Stage 3 | 4.5 (1.9-10.4) | 0.001 | 1 (0.4-3) | 0.968 |
| Stage 4 | 6.4 (4-10.1) | <0.001 | 4.5 (2.7-7.5) | <0.001 |
| Stage Unknown | 12.5 (8.3-19) | <0.001 | 6.8 (4.4-10.7) | <0.001 |
|  |  |  |  |  |
| Early Stage | 1.6 (0.8-3.1) | 0.198 | 1.2 (0.6-2.4) | 0.594 |
| Late Stage | 5.8 (3.9-8.7) | <0.001 | 3.3 (2.1-5.1) | <0.001 |
|  |  |  |  |  |
| Bone Connective Soft | 2.1 (1.1-4.3) | 0.033 | 0.9 (0.4-2.3) | 0.860 |
| Bowel | 1.7 (1.2-2.4) | 0.001 | 1.4 (1-2) | 0.029 |
| Breast | N/A | N/A | 0.1 (0-0.2) | <0.001 |
| Central Nervous System | 0.2 (0-1.7) | 0.154 | 1.4 (0.6-3) | 0.448 |
| Cancer of Unknown Primary | 5.1 (3.1-8.3) | <0.001 | 2.5 (1.5-4.3) | 0.001 |
| Gastro-Oesophageal | 5 (3.4-7.4) | <0.001 | 3.5 (2.2-5.7) | <0.001 |
| Head & Neck | 0.5 (0.2-1.2) | 0.125 | 0.4 (0.1-1.8) | 0.219 |
| Hepatobiliary | 4.7 (2.5-8.8) | <0.001 | 6.5 (2.9-14.2) | <0.001 |
| Leukaemia | 0.3 (0.2-0.6) | <0.001 | 0.2 (0.1-0.5) | <0.001 |
| Lung | 3.8 (3-4.9) | <0.001 | 2.4 (1.8-3.4) | <0.001 |
| Lymphoma | 4.7 (2.9-7.6) | <0.001 | 2.5 (1.4-4.2) | 0.001 |
| Melanoma | 0.2 (0-1.3) | 0.082 | 0.6 (0.2-1.7) | 0.333 |
| Myeloma | 1.2 (0.4-3.6) | 0.676 | 0.8 (0.2-2.8) | 0.693 |
| Other | 2.8 (0.8-9.9) | 0.110 | 1.5 (0.7-3.4) | 0.325 |
| Ovary | N/A | N/A | 1.8 (1-3.3) | 0.040 |
| Pancreatic | 8.6 (4.9-15.2) | <0.001 | 4 (2.4-6.6) | <0.001 |
| Prostate | 0.6 (0.4-0.8) | 0.003 | N/A | N/A |
| Renal Tract | 2.1 (1.4-3.2) | <0.001 | 1.4 (0.8-2.4) | 0.212 |
| Uterine | N/A | N/A | 1 (0.5-2) | 1.000 |

**Supplementary information 8b – women**

**
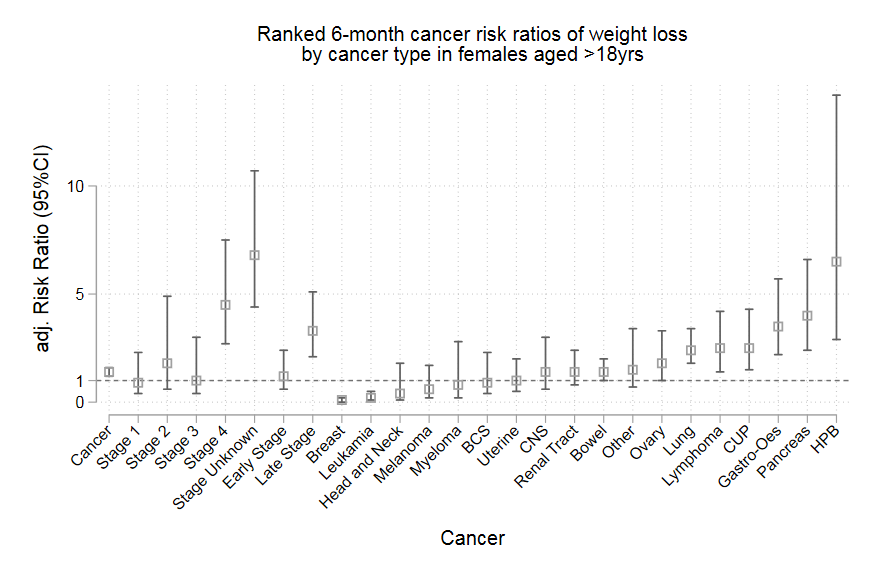
**

**Supplementary information 8c - men**

**
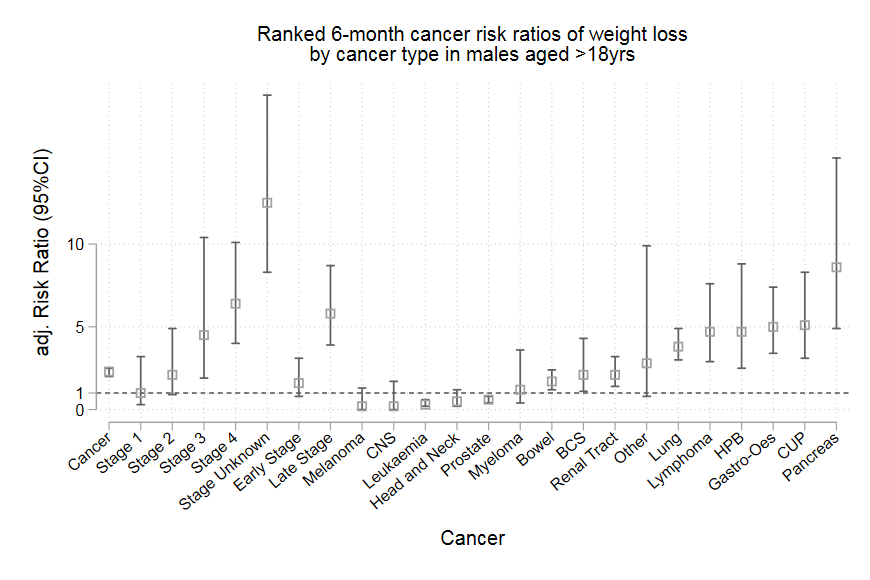
**
